# Qualitative grading of aortic regurgitation: a pilot study comparing CMR 4D flow and echocardiography

**DOI:** 10.1007/s10554-015-0779-7

**Published:** 2015-10-24

**Authors:** Raluca G. Chelu, Annemien E. van den Bosch, Matthijs van Kranenburg, Albert Hsiao, Allard T. van den Hoven, Mohamed Ouhlous, Ricardo P. J. Budde, Kirsten M. Beniest, Laurens E. Swart, Adriaan Coenen, Marisa M. Lubbers, Piotr A. Wielopolski, Shreyas S. Vasanawala, Jolien W. Roos-Hesselink, Koen Nieman

**Affiliations:** Department of Radiology, Erasmus MC, ‘s-Gravendijkwal 230, Room: Ca-207a, 3015 CE Rotterdam, The Netherlands; Department of Cardiology, Erasmus MC, Rotterdam, The Netherlands; Department of Radiology, University of California, San Diego, San Diego, CA USA; Department of Radiology, Stanford University, Stanford, CA USA

**Keywords:** Cardiac, Phase contrast, CMR 4D flow imaging, Eddy currents correction, Aortic regurgitation, Flow visualization

## Abstract

Over the past 10 years there has been intense research in the development of volumetric visualization of intracardiac flow by cardiac magnetic resonance (CMR). This volumetric time resolved technique called CMR 4D flow imaging has several advantages over standard CMR. It offers anatomical, functional and flow information in a single free-breathing, ten-minute acquisition. However, the data obtained is large and its processing requires dedicated software. We evaluated a cloud-based application package that combines volumetric data correction and visualization of CMR 4D flow data, and assessed its accuracy for the detection and grading of aortic valve regurgitation using transthoracic echocardiography as reference. Between June 2014 and January 2015, patients planned for clinical CMR were consecutively approached to undergo the supplementary CMR 4D flow acquisition. Fifty four patients (median age 39 years, 32 males) were included. Detection and grading of the aortic valve regurgitation using CMR 4D flow imaging were evaluated against transthoracic echocardiography. The agreement between 4D flow CMR and transthoracic echocardiography for grading of aortic valve regurgitation was good (κ = 0.73). To identify relevant, more than mild aortic valve regurgitation, CMR 4D flow imaging had a sensitivity of 100 % and specificity of 98 %. Aortic regurgitation can be well visualized, in a similar manner as transthoracic echocardiography, when using CMR 4D flow imaging.

**Electronic supplementary material:**

The online version of this article (doi:10.1007/s10554-015-0779-7) contains supplementary material, which is available to authorized users.

## Background

In the management of valvular heart disease assessment of transvalvular flow by cardiac magnetic resonance (CMR) can provide valuable incremental information to echocardiography, either as an alternative imaging modality in patients with poor acoustic windows, complex anatomy or for quantification of transvalvular flow. By current CMR practices, valvular flow patterns are measured across two-dimensional cross-sections, which are manually placed in the position of interest. Over the past 10 years there has been intense research in the development of volumetric visualization of intracardiac flow by CMR [1]. So-called CMR 4D flow imaging offers volumetric anatomical, functional and flow information during the entire cardiac cycle. It has several potential advantages over standard planar methods [2], including the ability to visualize complex flow patterns, select any plane when evaluating these CMR 4D flow datasets, without being limited to preselected planes and with the possibility to adapt the point of evaluation to the structural displacement throughout the cardiac cycle. Because all data is acquired during an uninterrupted ten-minute free-breathing scan, CMR 4D flow imaging is easy to perform and more comfortable for the patient. There are however a number of practical drawbacks that have prevented CMR 4D flow imaging from reaching the stage of widespread clinical implementation. While data acquisition was lengthy in the past, parallel imaging techniques [3, 4] have reduced the scan time to ten minutes or less. However, processing of the large data and correcting for eddy currents and gradient field distortions require dedicated software [5]. Previously published experiences were achieved by using in-house developed software for volumetric data pre-processing [6–10].

In this proof of concept study we evaluated the feasibility and performance of a cloud-based application that combines data pre-processing, including volumetric eddy currents correction, and visualization of CMR 4D flow data, and assessed its accuracy for the detection and grading of aortic valve regurgitation using echocardiography as reference.

## Methods

### Study population

Between June 2014 and January 2015 adult patients planned for clinical contrast-enhanced CMR were consecutively approached to undergo the supplemental CMR 4D flow examination, and prospectively included in the study (Table 1). The study was carried out in accordance with the principles of the Declaration of Helsinki and approved by the institutional Medical Ethics Committee. All participants gave written informed consent.

**Table 1:** Baseline characteristics of the study population

Characteristic	
Age (years)^a^	39 ± 15
Male gender	32 (59)
Body mass index (kg/m^2^)^a^	24.7 ± 5.2
Heart rate (beats/min)^b^	66 (48–208)
Sinus rhythm	51 (94 %)
Systolic blood pressure (mmHg)^a^	124 ± 14
Diastolic blood pressure (mmHg)^a^	77 ± 10
Clinical history	
Congenital heart disease	25 (46.2)
Aortic valve stenosis—corrected	4 (7.4)
Atrial septum defect	2 (3.7)
Bicuspid aortic valve	7 (12.9)
Coarctation—corrected	1 (1.8)
Marfan syndrome	1 (1.8)
Pulmonary valve stenosis—corrected	3 (5.5)
Tetralogy of Fallot—corrected	2 (3.7)
Turner syndrome	4 (7.4)
Ventricular septum defect—closed	1 (1.8)
Cardiomyopathies (CMP)	26 (48.1)
Anthracycline-induced CMP	1 (1.8)
Amyloidosis	1 (1.8)
Dilated CMP	4 (7.4)
Hypertrophic CMP	13 (24)
Non-compaction CMP	4 (7.4)
Sarcoidosis	3 (5.5)
Other	3 (5.5)
Heart transplant	1 (1.8)
Hypertension	1 (1.8)
Post-cardiac arrest	1 (1.8)

Unless otherwise specified, data are numbers of patients, with percentages in parentheses

^a^Data are means ± standard deviations

^b^Medians with minimum and maximum values

### Cardiac MR examination

The acquisition was performed using a 1.5 T or 3.0 T whole body scanners (Discovery MR450 and Discovery MR750, 45 mT/m, 200 T/ms, GE Medical Systems, Milwaukee, WI, USA) with a software version DV24.0, using dedicated 32-channel phased-array cardiac surface coils. Depending on the clinical request a variety of CMR examinations were performed including multi-planar cine steady-state free precession acquisitions, flow visualization using phase contrast sequence, contrast-enhanced angiography or delayed enhancement imaging.

### CMR 4D flow data acquisition

The CMR 4D flow data was acquired immediately, or shortly after the bolus injection of 0.1–0.2 mmol/kg gadolinium-based contrast agent (Gadovist 1 mmol/ml, Bayer, Mijdrecht, The Netherlands), depending on the clinical indication for contrast administration. The sequence was prescribed in the axial plane, with a matrix of 192 × 144 and 2.8 mm slices interpolated to 1.4 mm slices, while the entire thorax was included in the field of view during a free-breathing, ECG gated scan. Patients were also asked to breath regularly during the acquisition. The median views per segment was 4 and the median repetition time was 3.9 ms, resulting in a temporal resolution of 63 ms. When necessary, the views per segment and temporal resolution were adapted to limit the scan duration to approximately ten minutes (Table 2). Following recommendations from literature, the velocity encoding value was set at 250 cm/s, which is a good compromise for the 4D flow to allow acquiring low and high velocities simultaneously [11].

**Table 2:** Imaging parameters of the CMR 4D flow acquisition

Imaging parameter	Value
Repetition time (ms)^c^	3.9
Echo time (ms)^c^	1.5
Flip angle (°)	15
Acquired matrix size	192 × 160 × 78
Reconstructed matrix	256 × 256 × 156
Acquired spatial resolution (mm)	1.77 × 2.12 × 2.80
Reconstructed spatial resolution (mm)	1.33 × 1.33 × 1.40
Views per segment^a^	4 (3–5)
Temporal resolution (ms)^b^	62.8 (51–65)
Velocity encoding (cm/s)	250 × 250 × 250
Sampling	Poisson
Acceleration^c^	2.0 × 2.0
Median scanning duration (min:s)^b^	8:52 (7:37–9:50)

^a^Medians with minimum and maximum values

^b^Medians with interquartile range in parenthesis

^c^Medians

### Remote CMR 4D flow data correction, reconstruction and analysis

**Fig. 1:**
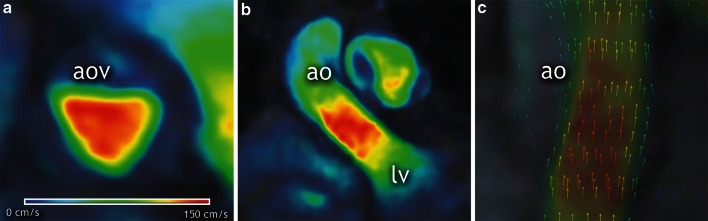
Aortic valve visualization with CMR 4D flow imaging. Short (**a**) and long-axis (**b**) cross-sectional views of the left ventricular outflow tract depicting systolic flow across the aortic valve. Because velocity-based *color-coding* lacks directional information, superimposed vectors display is used for confirmation of flow direction (**c**). The *color scale* can be manually modified as in this example where low velocities are displayed in *dark blue color* and equal or higher than 150 cm/s are displayed in *red color*. *lv* left ventricle, *ao* aorta

Because CMR 4D flow imaging is a phase contrast sequence, similar to planar flow acquisitions, magnitude images for anatomic information as well as images containing flow information may be reconstructed from the data. These large unreconstructed raw data sets (approximating 5 Gb per patient) were uploaded to a dedicated web-based software application (Arterys Inc., San Francisco, CA, USA) for data correction, visualization of the anatomical and flow components, and evaluation of the aortic regurgitation. As previously described [6], images were reconstructed for each cardiac temporal phase with a combined autocalibrating parallel imaging compressed sensing algorithm (L1-SPIRIT). Data was processed using large servers (Amazon Inc., Seattle, WA, USA). They are equipped with high frequency processors (Intel Xeon E5-2670, Intel Corporation, Santa Clara, CA, USA) and high-performance graphics processing units (NVIDIA Corporation, Santa Clara, CA, USA). The user logged into the application via internet connection from any type of computer or other device. Before the data sets were analyzed, the user could semi-automatically correct the images for encoding errors related to gradient field distortions and eddy currents. This approach is based on defining a threshold to identify regions with static tissue. These were then used to estimate eddy current induced varying phase offset errors, which were subsequently subtracted from the entire image [12]. Once the post-processing was complete and eddy currents correction performed the software facilitated interactive, real-time, manual navigation through the imaging volume using perpendicular multiplanar reformations and volume rendered reconstructions. The data could be reformatted into planes representing specific regions of interest (Fig. 1; Video 1). Flow could be displayed by color-coding of the velocity information or by vector rendering to display flow direction. For a red color on the color scale, a treshold of 150 cm/s was set.

### Evaluation of aortic valve function by CMR 4D flow imaging

The aortic valve was localized within the three dimensional anatomical dataset and displayed on three cross-sectional views that are perpendicular to each other. Using the cine display mode with superposition of flow onto the anatomy, the data was screened for the presence of reversed flow from the aortic valve during diastole. Identification of suspected aortic regurgitation was then evaluated using a temporally frame-by-frame approach. Because the velocity-based color-coded display of flow lacks directional information, the vector arrow display was used for confirmation (Fig. 1). Aortic valve regurgitation was defined as mild, moderate or severe. The grading criteria were adapted from echocardiographic recommendations [13, 14] using only the parameters available for both echocardiography and CMR 4D flow imaging: the ratios between the width of regurgitant jet and of the left ventricle outflow tract, and between the length of the regurgitant jet and of the left ventricle and the presence of reversal flow during diastole at the level of the descending aorta (Table 3). Two blinded readers (RGC and KN with 4 and 7 years of CMR experience, respectively) independently assessed each case and graded aortic regurgitation. A joint consensus reading served to solve discordant interpretations.

**Table 3:** Aortic regurgitation grading criteria

Grade	Ratio between the width of the regurgitant jet and the left ventricle outflow tract	Ratio between the length of the regurgitant jet and the left ventricle	Diastolic reversal flow in the descending aorta
Mild	<25 %	<25 %	Absent
Moderate	25–64 %	25–50 %	Trace
Severe	More than 65 %	More than 50 %	Present holodiastolic

Adapted from echocardiographic recommendations for assessment of native aortic valve regurgitation, only parameters available for both methods were used: the ratios between the width of regurgitant jet and of the left ventricle outflow tract, and between the length of the regurgitant jet and of the left ventricle and the presence of reversal flow during diastole at the level of the descending aorta [13, 14]

### Evaluation of aortic valve function by echocardiography

Two-dimensional transthoracic Doppler echocardiography was performed using a commercially available system (IE33, Philips Medical Systems, Best, The Netherlands). The gain settings were adapted to the patient’s characteristic to obtain the best quality of color Doppler. Because usually in aortic regurgitation the velocities are higher than 2 m/s, it is not possible to correct for aliasing phenomenon even by changing the aliasing velocity settings. Thus practically aliasing is always present. We set the aliasing velocity at 1.2 m/s.

Blinded to the CMR 4D flow imaging results, echocardiography images were independently evaluated by two cardiologists (AvdB and JR-H with 15 and 23 years of echocardiography experience, respectively), followed by a consensus reading. The presence of aortic regurgitation was graded using the same criteria as described above for the CMR 4D flow imaging. Because our paper focusses on the technical validation of 4D flow imaging we only considered parameters that could be assessed by both techniques.

### Statistical analysis

Statistical analysis was performed using SPSS (version 21 IBM, Armonk, NY, USA) and MedCalc (version 13.0; MedCalc Software, Ostend, Belgium). Categorical variables were reported as totals and percentages and continuous variables with a normal distribution as means ± standard deviations (SDs), or if data was skewed, as median with interquartile range. The diagnostic characteristics of CMR 4D flow imaging were evaluated against echocardiography by using C-statistics, including sensitivity, specificity, positive predictive value, negative predictive value with their corresponding 95 % confidence intervals (CIs), accuracy and Cohen’s kappa agreement test.

## Results

Out of 59 eligible patients, the scan failed in five because of insufficient data storage capacity on the MR scanner, leaving 54 patients for inclusion in the study. Median age of the population was 39 years (range 18–76), 32 were males and 46 % were known with congenital heart disease (Table 1). 69 % were scanned on 1.5 T and 31 % on 3.0 T MR scanner. No patients were excluded due to poor image quality of CMR 4D flow imaging or echocardiography. The median time between echocardiography and CMR 4D flow acquisition was 2 months.

### Performance of CMR 4D flow imaging

**Fig. 2:**
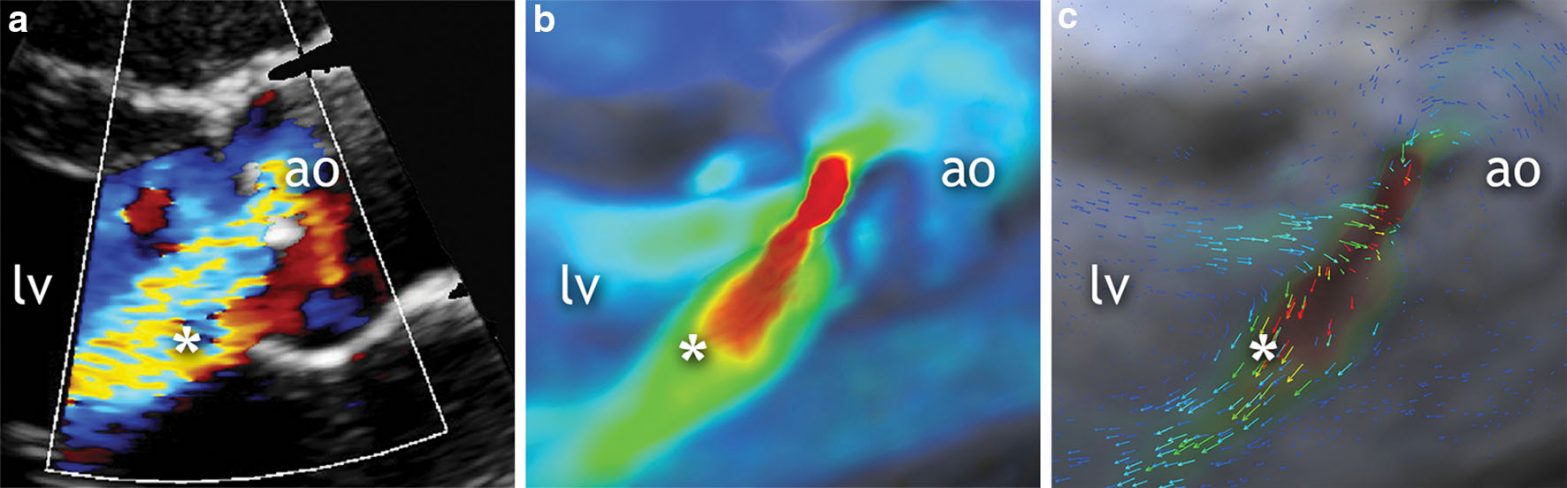
Aortic regurgitation by echocardiography and CMR 4D flow imaging. Case example of a 19-year-old man after balloon dilatation of the aortic valve for congenital aortic stenosis. Panel **a** Parasternal long axis view of aortic valve in diastole, showing moderate regurgitation (asterisk) demonstrated with color-flow Doppler echocardiography. Panels **b** and **c** Corresponding CMR 4D flow images showing the moderate aortic regurgitation. *Asterisk* regurgitant jet, *lv* left ventricle, *ao* aorta

**Fig. 3:**
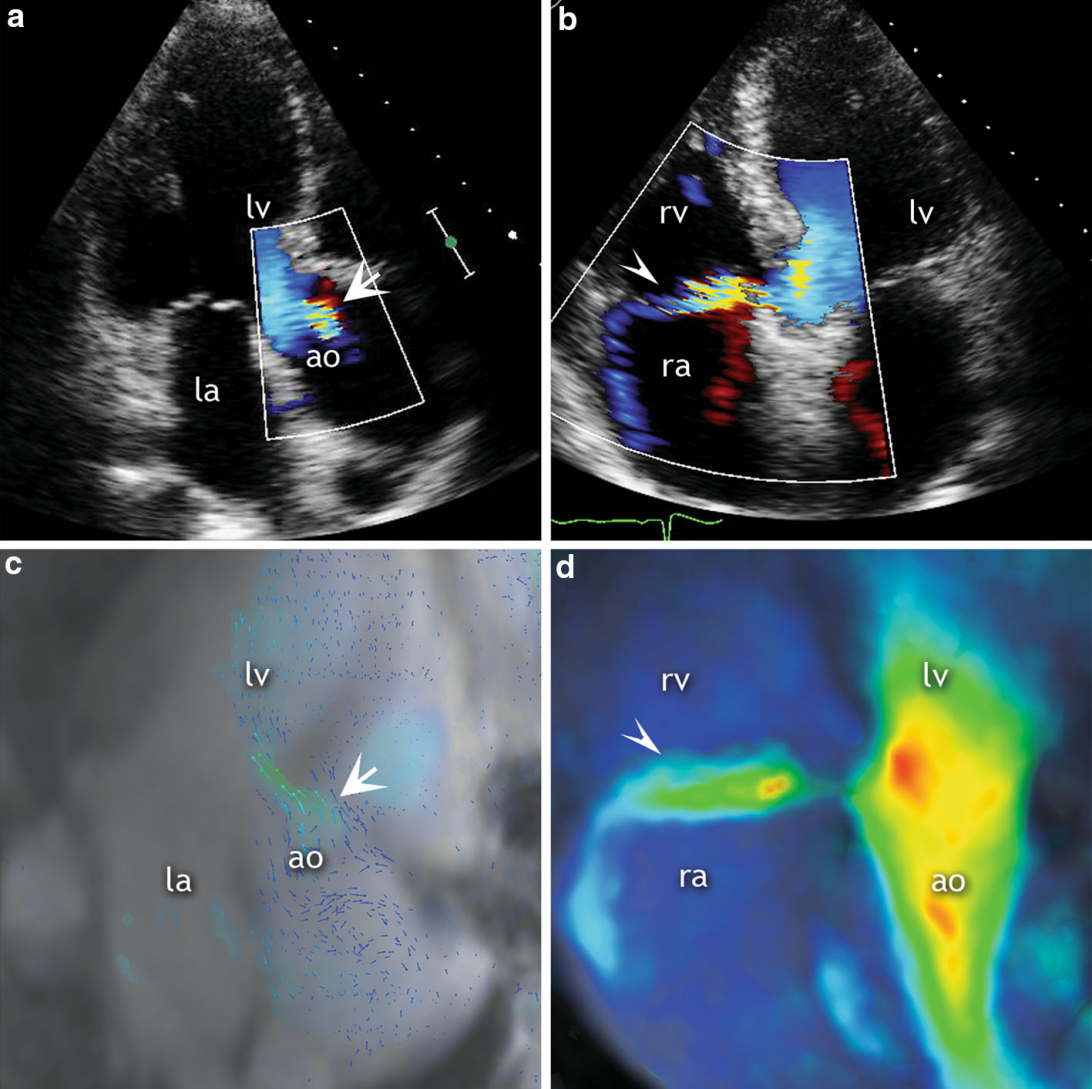
Mild aortic regurgitation and intracardiac shunt. A 47-year-old man post aortic valvotomy and Ross procedure for congenital aortic stenosis. Panel **a** Three chamber view with color-flow Doppler echocardiography showing a small aortic regurgitant jet (*asterisk*). Panel **b** Doppler echocardiography imaging, apical four chamber view, zoomed in on the basal septum and the right atrium; color flow through a typical Gerbode defect (*arrows*). Panels **c** and **d** CMR 4D flow imaging with corresponding views of the mild aortic regurgitation and the subvalvular, ventriculo-atrial jet, respectively. *lv* left ventricle, *ao* aorta, *ra* right atrium, *rv* right ventricle

The correction and visualization of the CMR 4D flow data and interpretation of aortic regurgitation required 10–15 minutes per patient. The agreement between CMR 4D flow imaging and echocardiography for the grading of regurgitation was good (κ = 0.73; Table 4). Representative case examples are shown in Figs. 2, 3 and Video 2. By CMR 4D flow imaging all four patients with moderate and 9 out of 15 patients with mild regurgitation were correctly identified. One case of mild regurgitation by echocardiography was interpreted as moderate by CMR 4D flow, and in 1 out of 35 cases CMR 4D flow identified mild regurgitation while echocardiography showed none. For the detection of any regurgitation, CMR 4D flow imaging demonstrated specificity of 97 % (95 % CI: 85–99 %), sensitivity of 74 % (CI: 49–91 %), positive predictive value of 93 % (CI: 68–99 %) negative predictive value of 87 % (CI: 73–96 %), accuracy of 88 % and κ = 0.74. To identify clinically relevant, more than mild regurgitation, CMR 4D flow imaging had sensitivity of 100 % (CI: 40–100 %), specificity of 98 % (CI: 89–100 %), positive predictive value of 80 % (CI: 29–97 %) and negative predictive value of 100 % (CI: 92–100 %), accuracy of 98 % and excellent agreement with echocardiography κ = 0.88.

**Table 4:** Agreement between CMR 4D flow imaging and echocardiography

CMR 4D flow imaging	Echocardiography			Total
	None	Mild	Moderate	
None	34	5	0	39
Mild	1	9	0	10
Moderate	0	1	4	5
Total	35	15	4	54

The correlation between the two methods, when assessing the aortic regurgitation, was κ = 0.73. When using a threshold of mild aortic regurgitation the correlation was κ = 0.74 and when using a threshold of moderate aortic regurgitation the correlation was κ = 0.88

## Discussion

In this study we report the first experiences with a cloud-based software application for pre- and post-processing CMR 4D flow data. We investigated its performance for the evaluation of aortic valve regurgitation. The novelty of this study is the use and validating of the online reconstruction and post-processing application. We selected aortic regurgitation for the validation study, because valvular regurgitation in particular benefits from 4D visualization. Except for the five cases where limited data storage capacity of the MR scanner caused difficulties, reconstruction and evaluation was successful in all other cases. For the detection and grading of aortic regurgitation, CMR 4D flow imaging correlated well with echocardiography and all patients with clinically relevant aortic regurgitation were correctly identified. There were two cases of overestimation, one mild aortic regurgitation graded as moderate with 4D flow and another one not visible with echocardiography which was graded as mild with 4D flow. There were also five mild regurgitations on echo, missed with 4D flow. We consider that CMR 4D flow is feasible and correlates well, but the population is not of sufficient size to conclude if there is systematic over- or underestimation. Out of the 5 underestimated regurgitations, two were scanned at 3.0 T and three at 1.5 T and from the two overestimated regurgitations one was scanned at 1.5 T and one at 3.0 T. Under the limitations of modest number of patients, we did not observe any indication that field strength was associated with accuracy.

In 2012, Hsiao et al. [6] used non-commercially available in-house software with a similar computational algorithm for data processing with color-speed overlay as in our paper, to evaluate valve regurgitation and intracardiac shunts with CMR 4D flow imaging against color Doppler echocardiography. The results are similar to our findings, good agreement (κ = 0.76) was achieved when applying a threshold of at least mild regurgitation, and also a substantial agreement (κ = 0.69) when using a threshold of more than mild regurgitation. The minor differences in agreement between both studies may be explained by the fact that in our study vector arrow overlay was used to help indicate the direction of the flow.

### Clinical utility of CMR 4D flow imaging

Echocardiography has been the primary imaging technique for the care of patients with structural heart disease, offering advantages in ease of performance, safety and cost. However, in complex disease and after surgery echocardiography alone is frequently insufficient. CMR offers an alternative imaging option that is helpful in a number of specific applications, e.g. imaging of myocardial perfusion or scar, flow quantification, or when echocardiography cannot be performed adequately due to technical reasons, such as insufficient acoustic window. By the current standard of care, CMR measures flow using two-dimensional, velocity-encoded sequences, and it displays flow perpendicular to a manually selected plane. It can be a lengthy process that requires good cooperation from the patient. Potential advantages of CMR 4D flow protocol are that anatomical, functional and flow information are obtained during a free-breathing acquisition of 7–10 minutes, which is more comfortable for the patient and easier to perform. Without a need to specify beforehand, or expert assistance during the examination, flow can be measured anywhere and in any direction within the great vessels of the thorax after the data has been acquired. While 2D sequences acquire flow information in a static plane, CMR 4D flow imaging allows for dynamic alignment of the plane of interest to the position of moving structures (e.g. valve annulus) [15]. Three-dimensional visualization of flow is also helpful for assessment of regurgitation jets that change direction during the heart cycle. However, clinical application of CMR 4D flow imaging is met by several technical challenges. Accurate representation of flow requires several pre-processing steps to correct for eddy currents and for magnetic field inhomogeneity. Experienced centers often rely on in-house developed solutions. To our knowledge this is the first report on the performance of a cloud-based software that applies volumetric eddy currents correction and direct, interactive flow visualization of CMR 4D flow data in a single tool package by internet connection, with remote processing of transferred raw datasets on high-performance workstations.

### Study limitations

This is a relatively small, single-center study. As a proof of concept we limited the analysis to the qualitative evaluation of aortic valve regurgitation. Because demonstration of technical feasibility for flow visualization was the aim of this study, only parameters available for both echocardiography and CMR 4D flow imaging were used for assessment of the regurgitation severity. The prevalence of aortic regurgitation was 35 %, though none of the patients had severe regurgitation. Future studies should expand into quantitative analyses, as well as a wider range of valve pathologies.

Future work is needed to determine optimal technical parameters for quantification of stenosis, regurgitation, shear stress, pressure gradients and other flow field derived metrics. To improve the contrast to noise ratio, we performed the CMR 4D flow acquisition after clinically-indicated intravenous contrast agent administration. Future work may focus of determining diagnostic accuracy without the use of contrast agent.

## Conclusion

In this study we showed that the use of a cloud-based reconstruction application with advanced eddy currents correction, integrated with interactive imaging evaluation tools allowed for remote visualization and interpretation of CMR 4D flow data and is sufficient for gross visualization of aortic valve regurgitation.

## Electronic supplementary material

Video 1 Aortic valve visualization with CMR 4D flow imaging. Short (Panel A) and long-axis (Panel B) cross-sectional views of the left ventricular outflow tract depicting systolic flow across the aortic valve. Because velocity-based color-coding lacks directional information, superimposed vectors display is used for confirmation of flow direction (Panel C).The color scale can be manually modified; in this example low velocities are displayed in dark blue color and equal or higher than 150 cm/s are displayed in red color. *lv* left ventricle, *ao* aorta (AVI 49171 kb)

Video 2 Aortic regurgitation by echocardiography and CMR 4D flow imaging. Case example of a 19-year-old man after balloon dilatation for congenital aortic stenosis. Panel A – Parasternal long axis view of aortic valve in diastole, showing moderate regurgitation (*asterisk*) demonstrated with color-flow Doppler echocardiography. Panel B and C – Corresponding CMR 4D flow images showing the moderate aortic regurgitation. *Asterisk* regurgitant jet, *lv* left ventricle, *ao* aorta (AVI 40235 kb)
